# Continental synchronicity of human influenza virus epidemics despite climactic variation

**DOI:** 10.1371/journal.ppat.1006780

**Published:** 2018-01-11

**Authors:** Jemma L. Geoghegan, Aldo F. Saavedra, Sebastián Duchêne, Sheena Sullivan, Ian Barr, Edward C. Holmes

**Affiliations:** 1 Department of Biological Sciences, Macquarie University, Sydney, New South Wales, Australia; 2 Marie Bashir Institute for Infectious Diseases and Biosecurity, Charles Perkins Centre, School of Life and Environmental Sciences and Sydney Medical School, The University of Sydney, Sydney, New South Wales, Australia; 3 Centre for Translational Data Science, The University of Sydney, Sydney, New South Wales, Australia; 4 Department of Biochemistry and Molecular Biology, Bio21 Molecular Science and Biotechnology Institute, University of Melbourne, Parkville, Victoria, Australia; 5 World Health Organization (WHO) Collaborating Centre for Reference and Research on Influenza, Melbourne, Victoria, Australia; 6 Department of Microbiology and Immunology, The University of Melbourne, Parkville, Victoria, Australia; 7 Faculty of Science and Technology, Federation University Australia, Gippsland Campus, Churchill, Victoria, Australia; Imperial College London, UNITED KINGDOM

## Abstract

The factors that determine the pattern and rate of spread of influenza virus at a continental-scale are uncertain. Although recent work suggests that influenza epidemics in the United States exhibit a strong geographical correlation, the spatiotemporal dynamics of influenza in Australia, a country and continent of approximately similar size and climate complexity but with a far smaller population, are not known. Using a unique combination of large-scale laboratory-confirmed influenza surveillance comprising >450,000 entries and genomic sequence data we determined the local-level spatial diffusion of this important human pathogen nationwide in Australia. We used laboratory-confirmed influenza data to characterize the spread of influenza virus across Australia during 2007–2016. The onset of established epidemics varied across seasons, with highly synchronized epidemics coinciding with the emergence of antigenically distinct viruses, particularly during the 2009 A/H1N1 pandemic. The onset of epidemics was largely synchronized between the most populous cities, even those separated by distances of >3000 km and those that experience vastly diverse climates. In addition, by analyzing global phylogeographic patterns we show that the synchronized dissemination of influenza across Australian cities involved multiple introductions from the global influenza population, coupled with strong domestic connectivity, rather than through the distinct radial patterns of geographic dispersal that are driven by work-flow transmission as observed in the United States. In addition, by comparing the spatial structure of influenza A and B, we found that these viruses tended to occupy different geographic regions, and peak in different seasons, perhaps indicative of moderate cross-protective immunity or viral interference effects. The highly synchronized outbreaks of influenza virus at a continental-scale revealed here highlight the importance of coordinated public health responses in the event of the emergence of a novel, human-to-human transmissible, virus.

## Author summary

We use a unique combination of large-scale laboratory-confirmed influenza surveillance and genomic sequence data to determine the pattern and determinants of influenza spread through time and space at a continental scale, utilizing 450,000 data entries gathered across Australia over a 10-year period (2007–2016). Our results reveal a remarkable epidemiological synchronicity across this large and geographically diverse continent, and in the face of enormous climatic variation, from the tropical north to the temperate south. Such synchronicity was especially marked during years associated with the emergence of antigenically distinct strains. This pattern of continental synchronicity highlights the importance of a highly coordinated responses in the event of the emergence of a novel, human-to-human transmissible, virus, and hence has broad-scale public health implications.

## Introduction

Seasonal and pandemic influenza remains one of the most important infectious diseases of humans and is associated with high levels of both morbidity and mortality [1]. Influenza epidemics occur annually due to the continual accumulation of small changes in surface antigens of influenza virus that escape host immunity and hence allow reinfection [2]. At present, four different forms of influenza virus co-circulate in human populations: the H3N2 and H1N1 subtypes of influenza A virus and the Victoria and Yamagata lineages of influenza B virus. Influenza-related mortality is highest in years when A/H3N2 viruses dominate, the rapid evolution of which also leads to the frequent emergence of antigenically distinct variants [2–4].

Influenza in temperate regions is characterized by an annual winter epidemic, whereas tropical regions experience less distinct annual patterns with sporadic outbreaks throughout the year [5]. Defining the seasonal and climatic drivers of influenza virus in both temperate and tropical regions has proven problematic and been the focus of much recent research [6, 7]. However, a detailed characterization of the spread of influenza virus through time and space at the scale of individual countries and/or continents has been hindered by the lack of spatially refined incidence data. Indeed, our understanding of influenza transmission has relied heavily on observations of influenza-like illness (ILI). Unfortunately, the accuracy of clinical diagnosis of influenza based on ILI alone is limited due to the considerable overlap of symptoms caused by other respiratory pathogens [8], with influenza virus reported to cause as few as 29% of all ILI infections [9]. Nevertheless, the detailed analysis of ILI data from the United States has shown that the nationwide spatial transmission of influenza virus is principally driven by workflow commuting [10]. In addition, both international and domestic air travel has been suggested as an important driver of influenza introduction and subsequent spread [11]. Much of this work suggests a high geographical correlation of influenza epidemics both at a local level in the United States [2, 10], and at the international level across the northern hemisphere [12].

The island continent of Australia offers a unique and opposing exploration of influenza dissemination. Australia’s human population is geographically concentrated in urban centers within two widely separated coastal regions that span over 4,000 kilometers. Hence, Australia is simultaneously one of the world’s most highly urbanized populations and least densely populated countries. The continent spans tropical to temperate latitudes and experiences highly variable climatic conditions. Human populations in temperate regions of Australia generally experience seasonal outbreaks of influenza infection between May and October [13]. Nevertheless, inter-seasonal influenza has been shown to persist locally with sustained transmission, particularly in tropical and sub-tropical regions such as Darwin and Townsville [6]. Hence, as well as antigenic variation, it is necessary to consider Australia’s climate complexity to inform its national influenza vaccination strategy [14].

The national and international spread of influenza virus is clearly complex, with the onset, duration and disease severity being largely dependent on the circulating virus strains, population immunity, human mobility and climatic factors. However, teasing apart the individual contributions of these factors has proven difficult, particularly given the diagnostic uncertainty associated with ILI data. Quantifying the spatiotemporal spread of influenza virus is particularly important for identifying the factors that contribute to its epidemic spread and for the precise targeting of control interventions. Here, we utilize large-scale laboratory-confirmed influenza incidence data collected by the National Notifiable Disease Surveillance System (NNDSS; http://www.health.gov.au/internet/main/publishing.nsf/content/cda-surveil-nndss-nndssintro.htm), which is under the auspices of the Communicable Disease Network Australia (CDNA), as well as viral genome sequence data collected on a global scale, to determine the correlation of influenza spread through time and space within Australia during the period 2007–2016. In particular, we sought to reveal the extent of epidemiological synchronicity within such a large and geographically diverse country, and what this means for understanding the determinants of influenza spread.

## Results

### Australian laboratory-confirmed influenza

The data provided by the Australian NNDSS are characterized by both spatial and temporal richness. The data set includes date of diagnosis, postcode of residence at the time of the test and, in the vast majority (98%) of cases, the influenza virus type detected. Although the overall aim for this surveillance is for complete case ascertainment, there are clearly some caveats in that only those cases for which health care was sought and a laboratory test conducted were represented. Nevertheless, this is one of the most well defined laboratory-confirmed influenza data sets at a nationwide level currently available globally.

The data set included the number of influenza cases per day between 2006 and 2016, by age group and virus type/subtype (S1 Fig). The majority (75%) of viruses were influenza A virus; however, in 70% of these cases the subtype was unspecified, with the remaining influenza A cases defined as H3N2 (8%) and H1N1 (22%). The number of un-subtyped influenza A virus cases varied among years. For this reason, we necessarily considered all influenza A cases in the main analysis as a single group rather than considering individual influenza A virus subtypes. Other viruses isolated included: A and B (0.01%); B (25%); C (0.0007%); and unknown (0.002%) cases. Data included the five-year age group of patients, which ranged from 0 to 85+ years old. In all years, the age group reporting the highest number of cases was the 0–4 year old group, with the exception of 2009 and 2015 in which the highest number of cases were reported from the 10–14 year old and 5–9 year old groups, respectively. Since data collection increased markedly from 2007 onwards, cases in 2006 and prior were excluded from further analysis.

### Time series of influenza viruses

**Fig 1:**
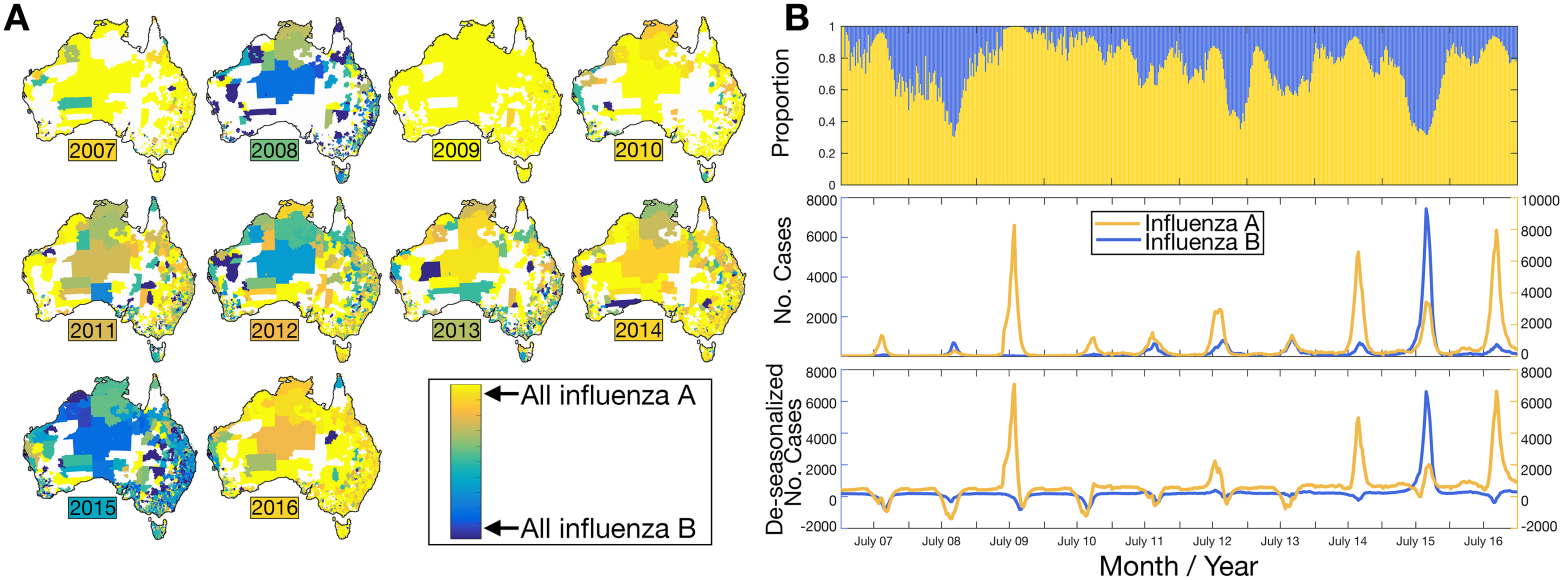
**(A)** Maps show the proportion of laboratory-confirmed cases of influenza A compared to influenza B between 2007–2016 for each Australian postal area corresponding to the adjacent color scale (i.e. blue: influenza B and yellow: influenza A). A colored box indicates the year, where the color denotes the overall national-level mean proportion for that year. Any deviation from this proportion suggests that influenza A and B were occupying different spatial regions. **(B)** (i) proportional time series of influenza A and influenza B cases; (ii) the number of laboratory-confirmed cases per week (left axis: influenza B; right axis: influenza A); and (iii) de-seasonalized number of laboratory confirmed cases, now showing only long-term trends and the noise components (left axis: influenza B; right axis: influenza A). For seasonal smoothing, data were de-trended and a stable seasonal filter was applied and subtracted from the time series data.

We examined the number of cases of influenza A and B, the most common influenza viruses in the data set, over the ten-year sampling time (Fig 1). Influenza C was not examined because of the small number of cases reported reflecting the fact that few laboratories in Australia currently test for influenza C. Maps show that the proportion of influenza A compared to influenza B varied each year between 2007–2016. As expected, very few influenza B cases were reported during the 2009 H1N1 pandemic, while in other years more locations reported higher proportions of influenza B cases. Influenza B dominated in 2015 with 60% of the total influenza cases, while only 39% were influenza A and the remaining cases were mixed infections of A and B. In all other years, influenza A was the dominant virus. During 2012, influenza A cases were concentrated in the southeast of Australia, while influenza B cases were largely concentrated in northern and western regions. While the majority of seasons displayed a co-circulation of influenza A and B, these two virus types often occupied and dominated different postcodes (Fig 1A). It is important to note, however, that many remote postcodes often reported very few cases each year, such that these patterns may be a reflection of low sample sizes in these locations. Despite this caveat, this spatial pattern might indicate that while one virus dominated in a particular geographic region, the other virus may have failed to establish, perhaps indicative of moderate cross-protective immunity or interference caused by the first establishing virus.

We next analyzed the time series of each influenza virus. These displayed a strong seasonal signature and an average annual peak at week ~34 (i.e. mid-August) for both influenza A and B, but was delayed in 2010 until week 39 for both viruses, likely due to the large-scale A/H1N1 outbreak during 2009. We also investigated the possibility of cross-protective immunity among influenza A and B by analyzing the time series in more detail (Fig 1, lower right panel). This reveals that, on occasion, there is a single dominant type annual peak during a seasonal cycle, again compatible with moderate cross-protective immunity or possibly residual immunity from previous seasons infection or vaccination.

### Establishment of influenza epidemics through time and space

**Fig 2:**
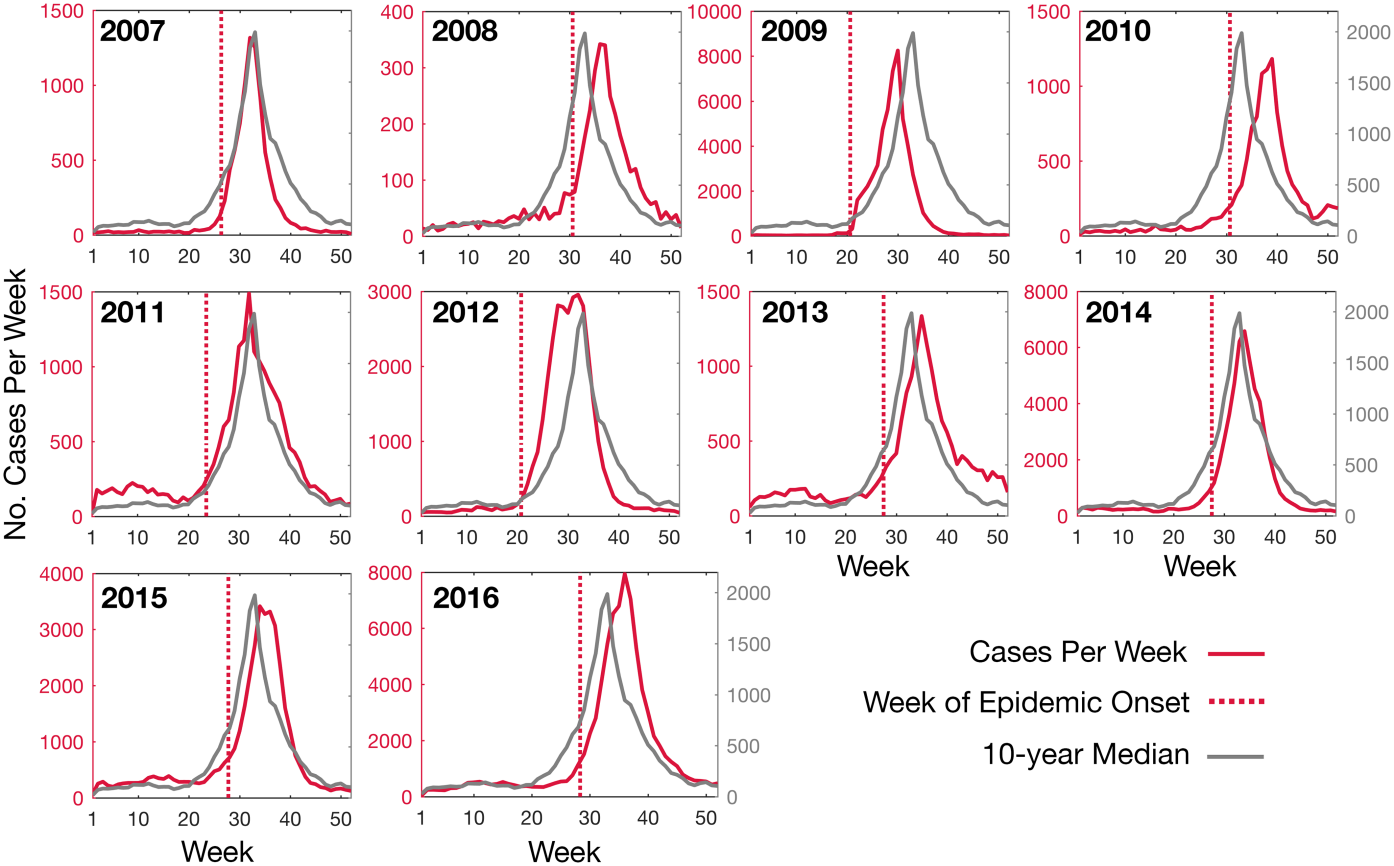
The number of laboratory-confirmed influenza A (all strains) cases per week for each influenza season from 2007 to 2016 (red line, left axes) and the ten-year median (grey line, right axes). The timing of ‘epidemic onset’ (vertical red, dashed line) for each year is shown.

We next estimated the timing of epidemic onset of influenza in Australia. To this end we first compared epidemic onset timing, which corresponds to the breakpoint in the piecewise regression, of influenza A across ten years from 2007–2016 (Fig 2). This comprised fitting linear regressions to the number of cases as a function of time, where the break-point marked the change in the slope of the line. Overall, the timing of the nationwide epidemic onset ranged from week 20.6 in 2009 to week 30.9 in 2010.

**Fig 3:**
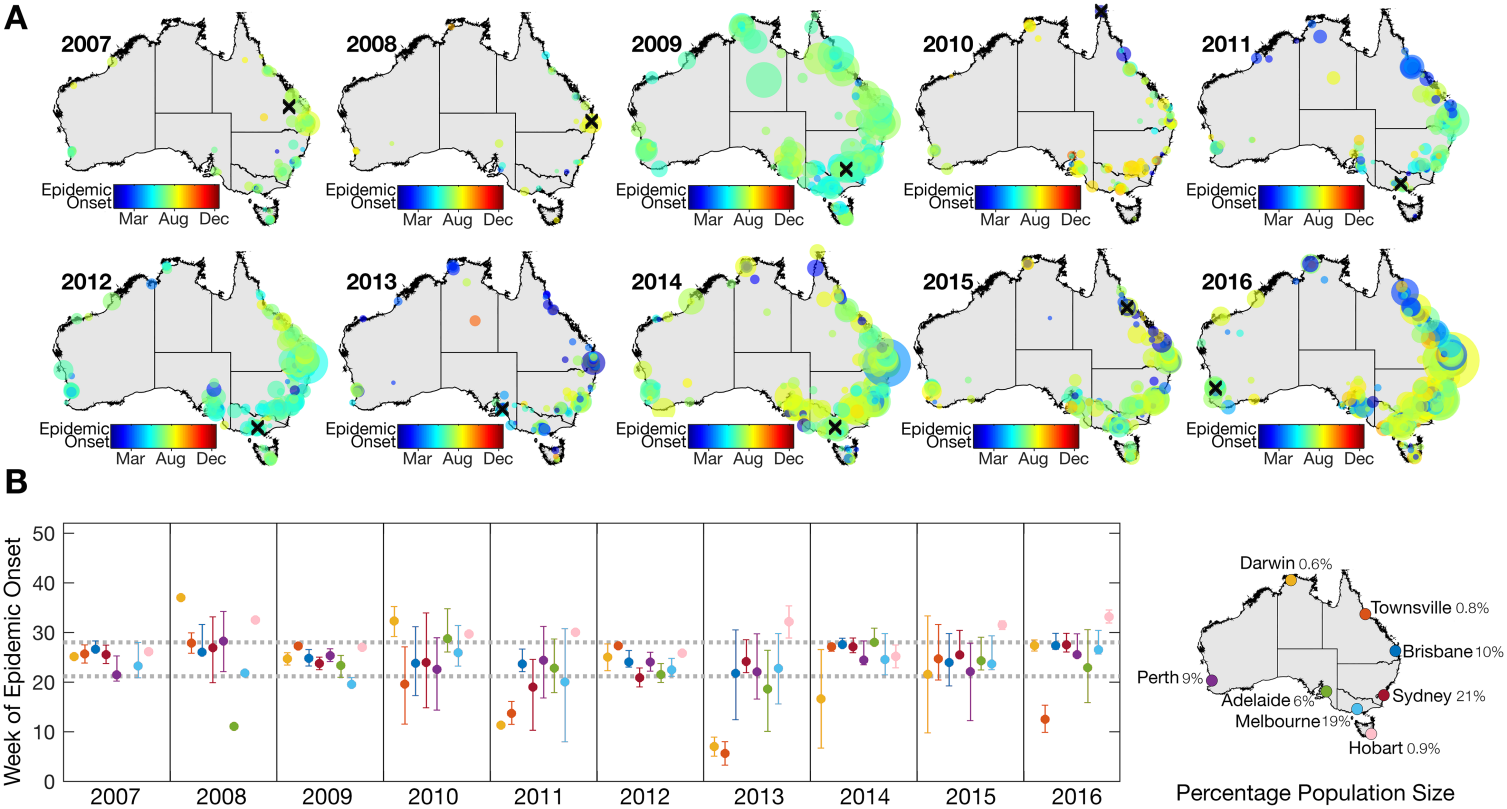
**(A)** Epidemic onset timing per location of laboratory-confirmed influenza A cases 2007–2016. Each circle on the map represents a distinct location (i.e. postcode) and the timing (week) of epidemic onset in each location is represented in color. The size of the circle represents the total number of cases in that location during that year. A black ‘X’ illustrates the location of the first sustained outbreak for each year. **(B)** The mean week of influenza A epidemic onset timing (as well as 25% and 75% quartiles) within metro postal areas in eight major cities: Darwin, Townsville, Brisbane, Sydney, Perth, Adelaide, Melbourne and Hobart. Dashed, grey horizontal lines indicate the 25% and 75% quartiles of overall onset times from these eight cities. The percentage of Australia’s population in each city is noted as of 2016.

To determine whether the timing of influenza outbreaks varied across Australia, we also estimated the epidemic onset timing for each sampling location. Estimation of epidemic onset timing required an established virus outbreak above baseline and is hence reliant on the strength of the influenza signal in each location. The total number of locations with established epidemics was therefore only a fraction of those locations with reported influenza cases, and ranged from 53 locations in 2008 to 679 locations in 2016 (Table 1). The time for influenza to reach all locations ranged from 28 weeks in 2009 to 43 weeks in 2013. Maps of epidemic onset timing show variable spatial patterns across all ten years (Fig 3A). In six out of ten years, the first established epidemic occurred in the southern cities (i.e. Melbourne, Adelaide, Canberra and Perth). In 2010, however, the first established outbreak was observed on Thursday Island in far north Queensland in the second week of January. Australia, which covers climatically diverse latitudes, often experiences sporadic yet sustained inter-seasonal outbreaks that have been shown to persist in tropical and sub-tropical regions [6]. Indeed, the data analyzed here show that epidemics in northern, tropical regions often precede and follow epidemics in more temperate regions (Fig 3B).

**Table 1:** Summary statistics describing the epidemic onset timing of laboratory-confirmed influenza A cases between 2007–2016 for each location sampled (i.e. Fig 3).

Year	Number of laboratory confirmed influenza A cases	Number of locations with reported influenza cases	Number of locations with established epidemics	Mean week of epidemic onset	Time for 50% of locations to be infected (weeks)	Time from 5th percentile of locations infected to the 95th percentile of locations infected (weeks)	Time from first location to be infected to last location to be infected (weeks)
2007	9149	1334	118	25.58	24.48	19.99	32.57
2008	3940	982	53	25.65	26.42	28.36	34.33
2009	55863	2032	534	23.38	20.89	9.91	28.52
2010	11684	1493	180	26.71	26.85	29.66	38.82
2011	19396	1661	285	21.41	21.36	27.81	38.31
2012	33705	1931	442	22.76	21.46	13.77	32.41
2013	17525	1646	288	21.16	21.88	31.69	42.94
2014	58755	2083	635	26.22	25.82	21.04	34.21
2015	39511	1934	505	23.99	24.53	27.4	35.97
2016	80979	2093	679	26.06	26.27	25.66	40.87

**Fig 4:**
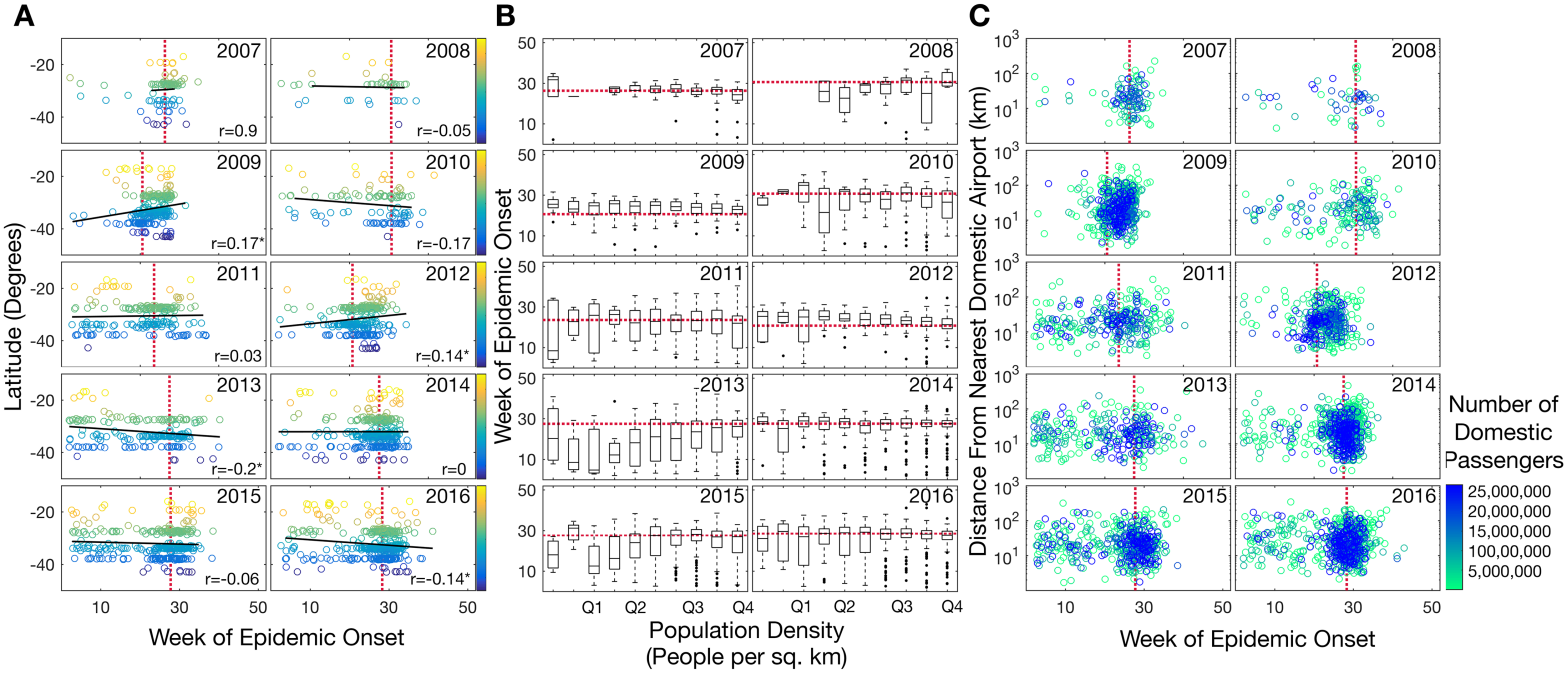
In each plot, a red, dashed line illustrates the overall annual epidemic onset time from Fig 2. **(A)** Latitude (degrees) as a function of epidemic onset time, showing only epidemics from postcodes located >145° longitude and hence covering the bulk of the Australian population. Pearson’s correlation coefficient is noted for each year, and an asterisk denotes *p*<0.05. **(B)** Boxplots of increasing population density within postcodes (people per square kilometer) and timing of the epidemic in that postcode. The x-axis gives quartiles of the densities, which ranged from 0.002 people per km^2^ to 12,877 people per km^2^. **(C)** The distance (km) from nearest airport as a function of epidemic onset time. Data points are colored by the total number of domestic passengers per year (see color bar).

During 2013 and 2016 there was a significant negative correlation between epidemic onset time and latitude; hence, northern regions experienced outbreaks that were, on average, earlier than southern regions (*p*<0.01). This was largely driven by isolated inter-seasonal outbreaks in Darwin and Townsville (Fig 3B). Conversely, the mean epidemic onset time in Hobart was consistently later than most other cities, although this may be due to the small sample size of metro postcodes in this area and was only significantly later in 2013 and 2016 (*p*<0.05). Overall, our analysis revealed that latitude was a poor predictor of epidemic onset time, and there was no consistent association even with those postcodes located along Australia’s east coast (i.e. >145° longitude), where most of the human population resides. Only in 2009 and 2012 did the onset of epidemics in southern latitudes precede those in northern latitudes (*p*<0.05) (Fig 4A).

There were no obvious radial patterns of virus dispersal from the location of the first outbreak each year. Rather, it was striking that epidemic onset was largely synchronized in the major cities, particularly during 2009 (Fig 3B). This result suggests dissemination of influenza through long-distance domestic air travel, likely coupled with multiple entries of influenza into Australia within a short period of time. We also found that onset timing of influenza A and B epidemics occurred around the same time each year, with the exception of the 2009 A/H1N1 pandemic season. In 2009, the mean epidemic onset timing of influenza B was week ~7 due to a few sporadic, inter-seasonal cases that preceded the emergence and takeover of the pandemic A/H1N1 (S2A Fig).

Although the important caveats regarding the sampling inconsistencies of influenza A subtypes (noted in the Materials and methods) prevented detailed analysis, both A/H1N1 and A/H3N2 also appeared to be largely synchronized in their epidemics, with the exception of 2009 and 2010; likely a consequence of the emergence of A/H1N1 pandemic strain in 2009 (S2B Fig).

To help reveal the determinants of influenza spread, we next investigated the role of population density and volume of air traffic between Australia’s domestic airports on the timing of epidemic onset. This revealed that there was no association between population density per postcode and the onset of epidemics (Fig 4B). Similarly, we found no linear relationship between the distance to the nearest domestic airport and the epidemic onset in a given postcode. Although the timing of epidemics was more clustered (i.e. synchronized) at postcodes close to airports with a higher number of domestic passengers (Fig 4C), busy domestic airports are located in major cities, which we have already shown to be well-synchronised with respect to epidemic onset (Fig 3B).

**Fig 5:**
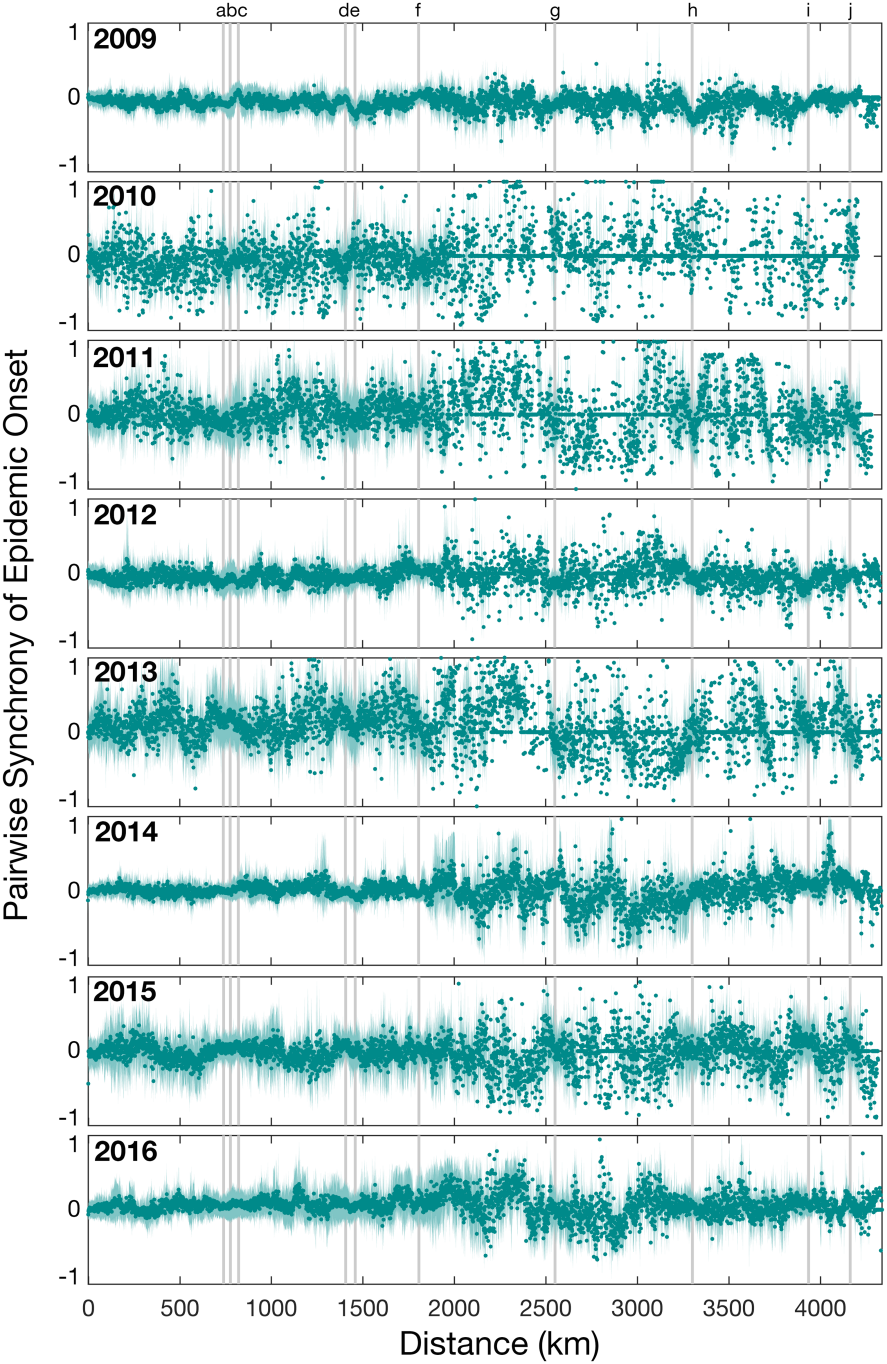
The normalized measure of pairwise synchrony in epidemic onset times between pairs of locations as a function of distance (kilometers). Values near zero indicate that epidemics start close together in time, while values near -1 and 1 indicate a lag in onset times. Each circle illustrates the mean pairwise synchrony for pairs within 1 kilometer and the shaded area represents the 95% percentile for the mean. Only years 2009–2016 are shown since these years had larger and more consistent sampling across all locations. Grey vertical bars mark the pairwise distances between the major cities: Sydney, Melbourne, Brisbane, Adelaide and Perth, in the order shown in S1 Table.

To determine the role of distance on the spatial spread of influenza in Australia, we investigated the difference in onset timing between pairs of locations and their pairwise distance (Fig 5). Spatial synchrony of epidemic onset timing was highly variable. In 2009, all locations experienced epidemic onset around the same time at, on average, week 23 with an interquartile range of week 21–26, suggesting very strong synchrony. In addition, epidemics were overall well-synchronized in 2012, 2014 and 2016 (all A/H3N2 dominant years) compared to other years. Importantly, epidemic onset timing was not correlated with geographic distance. In fact, the furthest points tended to display synchrony in onset timing suggesting that influenza reached both coastal extremes of Australia at approximately the same time. For example, across all years sampled, there was no significant difference in epidemic onset timing between Sydney and Perth, which are separated by a distance of 3,300 kilometers. In addition, points that fall on the grey vertical bars seem to be close to zero, meaning that there is little difference in onset time between pairwise locations. Although there appears to be a synchrony break at about 1750–2000 km, this reflects the fact that there are few populous postcodes that have this range of pairwise distances. Overall, these results suggest a highly synchronized epidemic onset timing within the most populous Australian cities that may be enhanced during years with antigenic novelty.

### Global circulation and introduction of influenza virus into Australia

**Fig 6:**
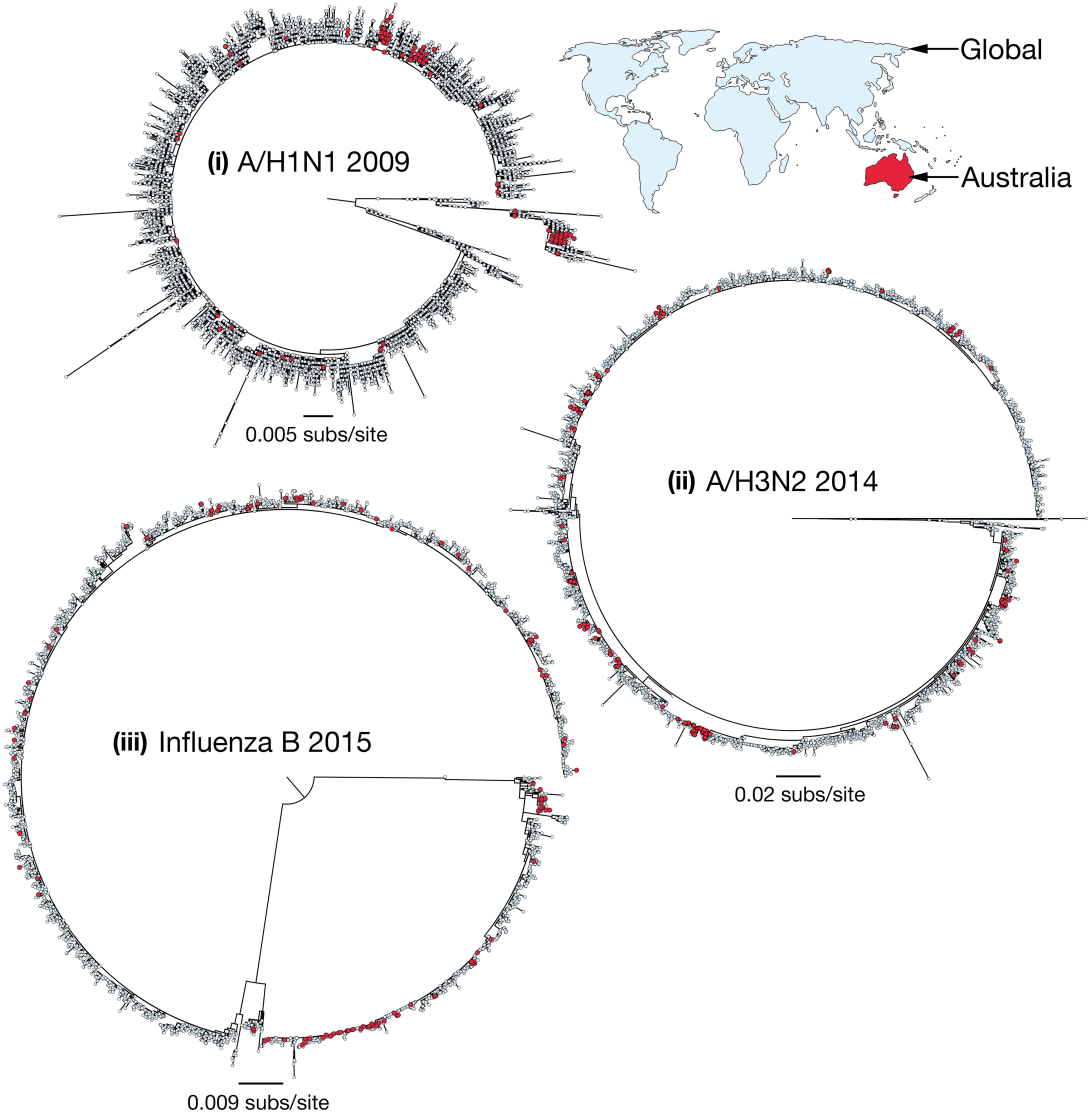
Maximum likelihood phylogenetic trees of global influenza virus. All available HA genetic sequences were downloaded from the GISAID EpiFlu database (platform.gisaid.org) for the following years and subtypes: (i) 2009 H1N1 (n = 10,016); (ii) 2014 H3N2 (n = 5,584); and (iii) 2015 Influenza B (n = 3,882). Sequences that were isolated from Australia are highlighted in red. More detailed analysis of patterns of spread within Australia are shown in S3 Fig.

To determine the relationship of those influenza viruses present in Australia and those circulating globally, we inferred phylogenetic trees for three subtypes over key years: (i) influenza A H1N1 during 2009; (ii) influenza A H3N2 during 2014; and (iii) influenza B during 2015 (Fig 6). Because of their relatively large data sets, we utilized global hemagglutinin (HA) gene sequence data comprising 19,482 sequences and analyzed the phylogeographic patterns present in the data, particularly the positions of the Australian sequences. For each of these influenza seasons there was little clustering of Australian sequences. Indeed, Australian sequences were dispersed across all the phylogenies, indicating multiple introductions into Australia from the global population as is common in other localities [15]. For example, the 224 Australian sequences available from the outbreak of H1N1 influenza A virus in 2009 fell into 41 phylogenetically distinct clusters or single lineages. The largest clades comprising Australian-only sequences were (i) H1N1, 2009 = 52 (of 224 (23%) Australian samples); (ii) H3N2, 2014 = 23 (of 196 (12%) Australian samples); and (iii) influenza B, 2015 = 30 (of 164 (18%) Australian samples). In addition, these Australian clades were sampled from many different locations within Australia, highlighting the importance of virus spread within Australia (S3 Fig) as noted previously [6]. For example, in 2014, A/H3N2 appears to enter the state of Victoria and from this location spreads to both the east (i.e. Sydney, Brisbane and Newcastle) and the west (i.e. Perth) coasts (S3C and S3D Fig; note that only sequences with known sampling locations within Australia are shown).

## Discussion

We examined the spatial and temporal spread of influenza viruses in Australia over ten years between 2007–2016 using a unique data set of spatially refined laboratory-confirmed influenza cases that comprises >450,000 entries. While many studies of the spatial spread of influenza are based on ILI data, in which accuracy is often highly variable and not well measured, our data set offers a rare opportunity to study laboratory-confirmed influenza (based mostly on real-time PCR testing). We focused on the onset time of established epidemics in each postal region (by postcode) across Australia in which data were reported. Importantly, establishment of an epidemic requires sustained transmission compared to a baseline number of cases, because the number of cases at baseline may not necessarily be associated with a transmission event leading to an epidemic. Detection of established epidemics was therefore reliant on virus outbreaks *above* baseline and thus the strength of the epidemic signal in each location.

The most prominent result of our study was that despite its huge climatic variation sustained influenza epidemics in Australia were often highly synchronized, especially during years that were observed to be associated with antigenically distinct strains. Although we were unable to assess pairwise synchrony by A subtype or B lineage (due to lack of data), we observed that the onset of nationwide epidemics was more synchronized during years in which there was emergence of novel strains or distinct antigenic changes, including 2009 (pandemic A/H1N1), 2012 (a new A/H3N2 variant) and 2014 (another new A/H3N2 variant). However, due to the low level of exact influenza A subtype data we could not assess whether the pairwise synchrony was entirely driven by these subtypes. For example, although 2009 and 2012 were dominated by A/H1N1 and A/H3N2, respectively, more precise subtype data suggested that both A/H1N1 and A/H3N2 viruses were present, with A/H1N1 circulating early in the season and A/H3N2 circulating later in the season [16]. Despite the obvious importance of domestic travel in driving the spread of influenza, it is likely that onset synchronicity has been enhanced by the multiple introductions of global influenza viruses into Australia, a process that was clearly apparent in our large-scale phylogeographic analysis. We also believe that this pattern is robust to ascertainment bias. In particular, although the rate of spread during 2009 was much higher compared to other years, the mean week of epidemic onset across all locations was later, at 23 weeks, compared to 21 weeks in 2011 and 2013 (Table 1). In addition, that all years studied exhibited greater nationwide synchrony than previously observed in the US [10] suggests that the pattern is indeed genuine and reflects the intrinsic dynamics of influenza in Australia. This synchronicity occurs despite the diverse array of climate types present across the Australian continent, suggesting that such seasonal epidemic dynamics override considerable climatic variation.

The onset of epidemics during the 2009 influenza season exhibited the greatest synchrony. Famously, 2009 was dominated by the emergence of a novel influenza A/H1N1 virus that resulted in a global pandemic and displaced the previous lineage of A/H1N1 that had circulated in the human population since 1977. We found that the time from the 5^th^ to the 95^th^ percentile of locations to be infected was less than 10 weeks in 2009, compared to an average time of ~25 weeks for the other years studied here. In Australia, up to 65% of ILI clinical isolates tested positive for influenza A and by early July 2009, A/H1N1 accounted for 90% of influenza A isolates [17]. Indeed, following its initial detection in the United States in April 2009, pandemic A/H1N1 rapidly spread and genetically diversified throughout the global human population [18].

Influenza seasons in 2012, 2014 and 2016 were also characterized by highly synchronous epidemics and were dominated by A/H3N2 viruses, and often associated with distinct antigenic changes. Specifically, 2012 saw the emergence of A/Victoria/361/2011-A/Texas/50/2012-like viruses (3C.1 clade), and in 2014 the emergence and co-circulation of three genetically distinct viral clades; the A/Hong Kong/4801/2014-like viruses (also referred to as 3C.2a clade), A/Switzerland/9715293/2013-like viruses (3C.3a) and A/Newcastle/22/2014-like viruses (3C.3b). Following both influenza seasons, there was a change in the H3 vaccine recommendation for the following year (2013 SH vaccine: A/Victoria/361/2011-like virus and for 2015 SH vaccine: A/Switzerland/9715293/2013-like virus) [19]. In addition in 2016, a highly prevalent new clade (3C.2a1) also circulated. Although no major antigenic changes were detected that were distinct from the Hong Kong/4801/2014-like viruses that had circulated in 2015, 2015 was dominated by influenza B such that relatively few A/H3N2 cases were detected compared to previous years. Consequently, the population was less exposed to A/H3N2 during 2015, likely leading to lower levels of population immunity in years immediately following.

We found a distinct lack of radial spread from the first point of virus entry; instead, populous cities tended to have well-synchronized epidemics. While data on work flow patterns in Australia were unavailable for this study, this national synchrony suggests that transmission patterns driven by work flow may have played a relatively minor role in influenza epidemic spread. Rather, these synchronized epidemics across Australian cities suggest rapid dissemination either through domestic flight traffic and/or by multiple global introductions. Indeed, our phylogeographic analysis of global influenza gene sequence data from key years shows that there have been multiple introductions of influenza into Australia annually, which were then able to establish transmission chains across the country, similar to that seen in other localities [20]. These continual introductions from the global population are emphasized by the relatively small number of clades exclusively comprised of Australian isolates. Overall, this pattern again highlights the fluidity with which influenza viruses spread both nationally and globally [21], and which acts to give annual epidemics a distinct degree of synchrony.

The apparent lack of radial virus dispersal observed in Australia also suggests that short-range commuter transmission has not played a major role in epidemic spread, or that it cannot be resolved in the scale of the data analyzed here. This sits in marked contrast to previous studies of more homogeneously-populated countries such as the United States, in which the analysis of ILI data revealed that virus dispersal was predominantly localized with distinct radial patterns from an infected location, with the estimated risk of transmission decreasing sharply with geographic distance [10]. Although it is possible that some of this difference reflects underlying differences in the data collected (i.e. laboratory-confirmed influenza versus ILI), the degree of synchronicity that we have observed during novel antigenic influenza seasons supports continued highly coordinated public health responses across Australia’s populous cities in the event of the emergence of a novel, directly transmissible, virus. Accordingly, in light of the speed with which novel viruses spread, the most populous cities experience synchronized epidemics, likely to be driven by strong domestic connectedness and international travel. These observations may be used to inform prospective pandemic planning efforts both in Australia and likely in other highly urbanized localities.

## Materials and methods

### Data collection

Laboratory-confirmed influenza notifications data were requested from CDNA. These data can be requested from CDNA (http://www.health.gov.au/cdna), pending appropriate human research ethics committee approval and from the data custodians in each jurisdiction. Ethical approval for this project was granted by The University of Sydney, project number 2015/625.

Laboratory-confirmed influenza is a notifiable disease in Australia, with notifications made by health care professionals and laboratories to jurisdictional health departments. The data set included 454,800 entries from 2,510 distinct postal areas, collected between 1^st^ January 2006 and 31^st^ December 2016. These entries included the patient’s age (within a five-year age group) and, in 98% of cases, the influenza virus type (i.e. A, B or C) detected. The ‘diagnosis date’ represented either the onset date or, where the date of onset was not known, the specimen collection date or the notification date. Data collected in 2006 were excluded from further analysis since data collection in all locations became more consistent from 2007. This left a final data set size of 451,480 entries spanning ten years collected between 2007 and 2016.

### Time series analysis

We first analyzed the number of laboratory-confirmed influenza cases of influenza viruses A and B to determine the dominant viruses present in Australia between 2007–2016. All cases specified as influenza A and B were included in this analysis. Only 33% of influenza A notifications included the subtype (i.e. A/H1N1 or A/H3N2), precluding further analysis by subtype. Since ‘diagnosis date’ might represent the time from the onset of symptoms or the date at which the specimen was collected, we aggregated the data by the number of cases per week and thus considered the weekly time series of these laboratory-confirmed cases. We explored both the proportion and number of cases of each virus across Australia. For seasonal smoothing, data were de-trended and a stable seasonal filter was applied and subtracted from the time series data (using Matlab v.2016b). By removing the seasonality of the time series, only the long-term trends and the noise components of the data were exposed.

### Epidemic onset timing

We estimated the ‘epidemic onset timing’, defined as the timing of the break-point in influenza incidence [10], across the ten-year period for which sufficient incidence data were available (i.e. 2007–2016). For each year, we therefore fitted piecewise linear models to determine the break-point in influenza incidence using the *Segmented* package in R [22]. Importantly, the break-point represents the time at which an epidemic can be considered established–in other words, the time of epidemic onset–rather than the time at which the virus first entered Australia each year. The establishment of an epidemic requires the sustained transmission of influenza, whereas an introduction simply represents the first case of influenza each year regardless of whether that first introduction triggered an outbreak. To this end, the onset timing of all influenza A (regardless of subtype) epidemics for each year was determined since these represented the majority of cases in the data set.

With these data in hand we aimed to better understand the spatial and temporal spread of influenza in Australia. To this end we estimated the epidemic onset timing in each postcode area for all cases of influenza A between 2007–2016. We investigated the synchrony of epidemic onset timing between pairs of locations and their pairwise geographical distance within a 1 kilometer range. Finally, we compared the epidemic onset timing between major cities in Australia (Sydney, Melbourne, Brisbane, Adelaide, Townsville, Darwin, Hobart and Perth), and between laboratory-confirmed influenza A and B viruses.

Next, we performed additional quantitative analyses of the strength of association between particular socio-economic parameters and epidemic onset timing. First, we investigated the role of population density within postcodes and the timing of epidemic onset. Accordingly, population size per postcode (taken from the 2016 Australian census data available at http://www.abs.gov.au/census) and postcode geographic data (available from the Australian Bureau of Statistics; http://www.abs.gov.au/ were used to calculate population density (i.e. number of people per square kilometer). Second, to explore the association between the extent of air travel and epidemic onset, we calculated the number of domestic passengers on inbound and outbound flights from the busiest 96 airports in Australia (data obtained from https://bitre.gov.au/statistics/aviation/), as well as the distance (in kilometers) from each postcode to its nearest airport.

### Phylogeography of influenza virus

To further investigate the introduction and subsequent spatial spread of influenza virus in Australia, particularly the number of introductions in any one influenza season and the presence of Australia-specific clades, we estimated the phylogenetic relationships of influenza virus A and B on a global scale. For this we utilized global gene sequence data available from the GISAID EpiFlu database (platform.gisaid.org) [23, 24] (note that these data are unlinked to the influenza incidence data analyzed here). All available HA genetic sequences, with a minimum length of 500 nucleotides, were downloaded for years in which global influenza data were abundant: (i) 2009 H1N1 (n = 10,016); (ii) 2014 H3N2 (n = 5,584); and (iii) 2015 influenza B virus (n = 3,882). Each sequence data set was aligned using the multiple-sequence alignment method available in the MAFFT program, using the FFT-NS-1 strategy [25]. Phylogenetic inference utilized the maximum likelihood (ML) method available in RAxML (v8.2.10) [26], applying the general time reversible (GTR) nucleotide substitution model with a gamma (Γ) distribution of among-site rate variation. Support for individual nodes was assessed using a bootstrap procedure with 100 replicates and phylogenetic trees were annotated in FigTree (v1.4.3).

## Supporting information

S1 Table The order of grey vertical bars in Fig 4 in the main text that mark the pairwise distances between the major cities. (DOCX)

S1 Fig Summary of the data set. The number of laboratory-confirmed influenza cases in Australia, per day, from 1^st^ January 2006 until 31^st^ December 2016, showing **(A)** patient age ranges; and **(B)** the type/subtype of the virus detected.
(TIF)

S2 Fig **(A)** The mean (and 95% confidence intervals) week of epidemic onset timing for influenza A (both subtypes and unsubtyped data) and B in Australia. **(B)** The mean (and 95% confidence intervals) week of epidemic onset timing for influenza A/H1N1, A/H3N2 and B.
(TIF)

S3 Fig Maximum likelihood phylogenetic trees of influenza virus, showing the two largest clades isolated from Australia that had specific sampling location available from each year shown in Fig 6 in the main text (A-B: A/H1N1 2009; C-D: A/H3N2 2014; E-F: influenza B 2015). HA genetic sequences were downloaded from the GISAID EpiFlu database (platform.gisaid.org). Trees are rooted as in Fig 6.
(TIF)
