# Correction: Continental synchronicity of human influenza virus epidemics despite climactic variation

**DOI:** 10.1371/journal.ppat.1006903

**Published:** 2018-02-07

**Authors:** Jemma L. Geoghegan, Aldo F. Saavedra, Sebastián Duchêne, Sheena Sullivan, Ian Barr, Edward C. Holmes

In the title of this article, the word “climactic” should be “climatic”. The correct title is: Continental synchronicity of human influenza virus epidemics despite climatic variation. The correct citation is: Geoghegan JL, Saavedra AF, Duchêne S, Sullivan S, Barr I, Holmes EC (2018) Continental synchronicity of human influenza virus epidemics despite climatic variation. PLoS Pathog 14(1): e1006780. https://doi.org/10.1371/journal.ppat.1006780
