# Supplementary material for: Continental synchronicity of human influenza virus epidemics despite climactic variation
Source: PLoS Pathog. 2018 Jan 11;14(1):e1006780. doi: 10.1371/journal.ppat.1006780 (PMC5764404; doi:10.1371/journal.ppat.1006780)
Supplement: S1 Table — (DOCX) [file ppat.1006780.s001.docx]

**Supplementary Table 1.** The order of grey vertical bars in Figure 4 in the main text that mark the pairwise distances between the major cities.

|  | Sydney | Melbourne | Brisbane | Adelaide |
| --- | --- | --- | --- | --- |
| Melbourne | b |  |  |  |
| Brisbane | c | d |  |  |
| Adelaide | e | a | f |  |
| Perth | j | i | h | g |
