# Supplementary figures and images for: Continental synchronicity of human influenza virus epidemics despite climactic variation
Source: PLoS Pathog. 2018 Jan 11;14(1):e1006780. doi: 10.1371/journal.ppat.1006780 (PMC5764404; doi:10.1371/journal.ppat.1006780)

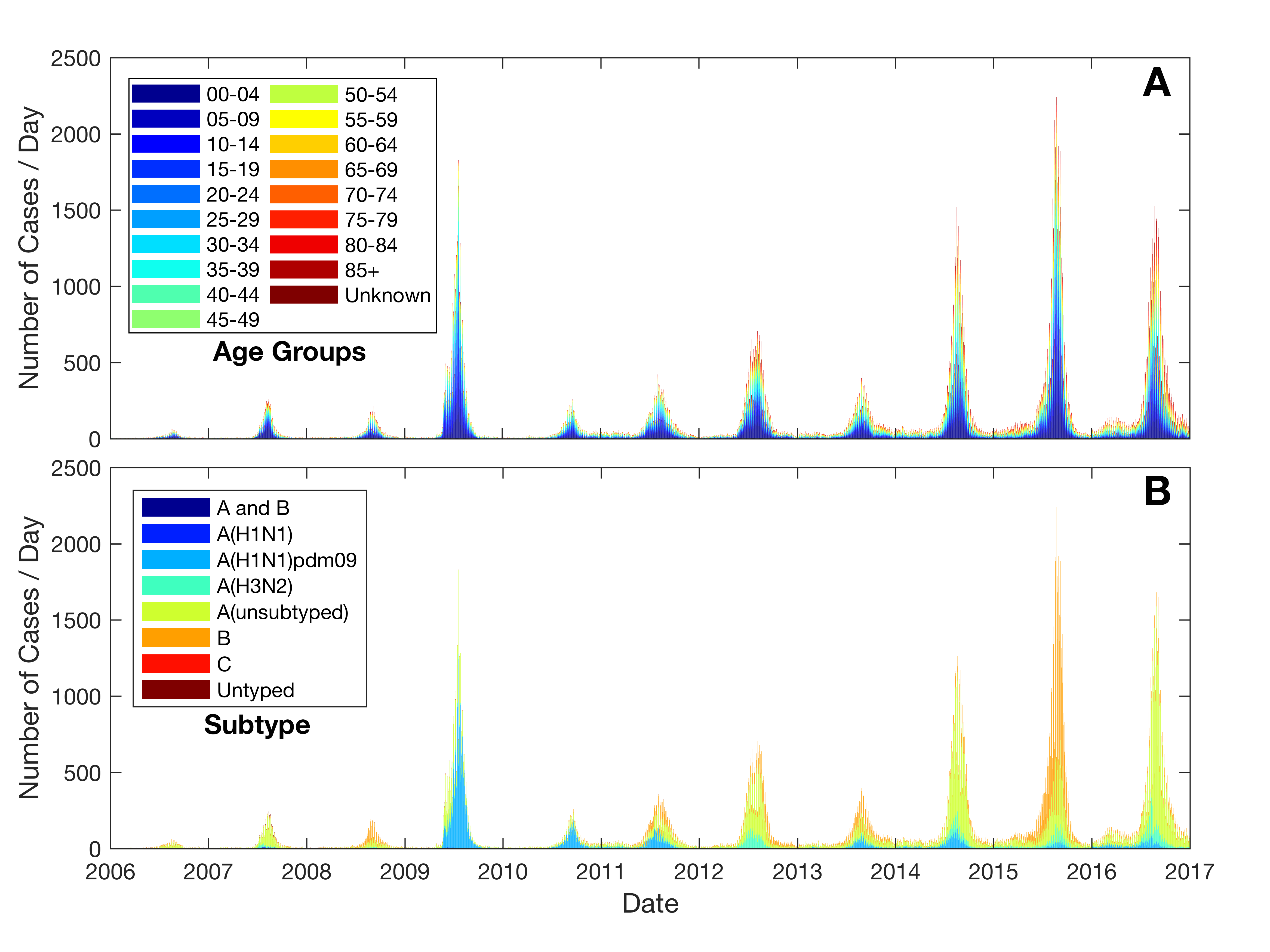

Supplement: S1 Fig — The number of laboratory-confirmed influenza cases in Australia, per day, from 1st January 2006 until 31st December 2016, showing (A) patient age ranges; and (B) the type/subtype of the virus detected. (TIF) [file ppat.1006780.s002.tif]

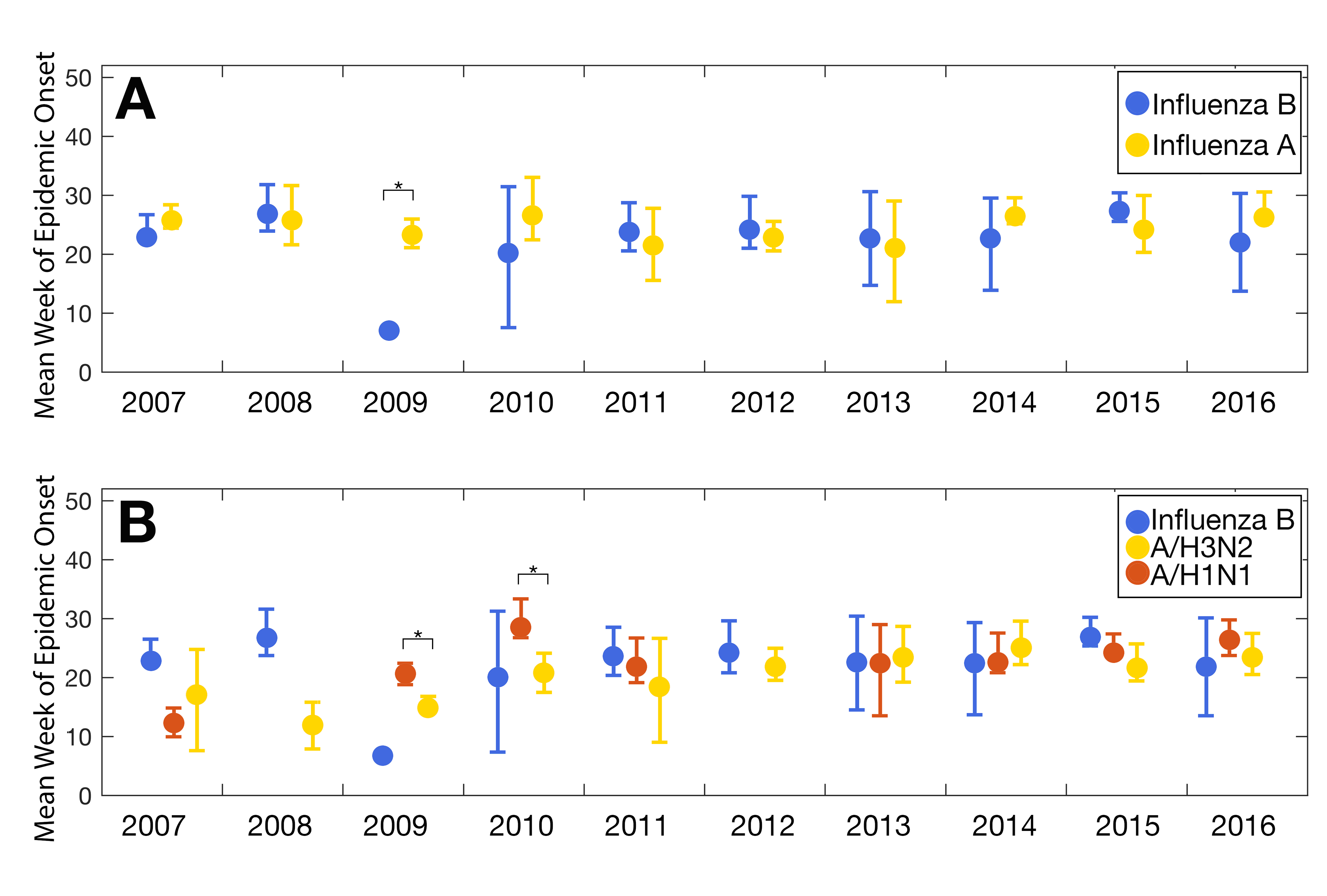

Supplement: S2 Fig — (A) The mean (and 95% confidence intervals) week of epidemic onset timing for influenza A (both subtypes and unsubtyped data) and B in Australia. (B) The mean (and 95% confidence intervals) week of epidemic onset timing for influenza A/H1N1, A/H3N2 and B. (TIF) [file ppat.1006780.s003.tif]

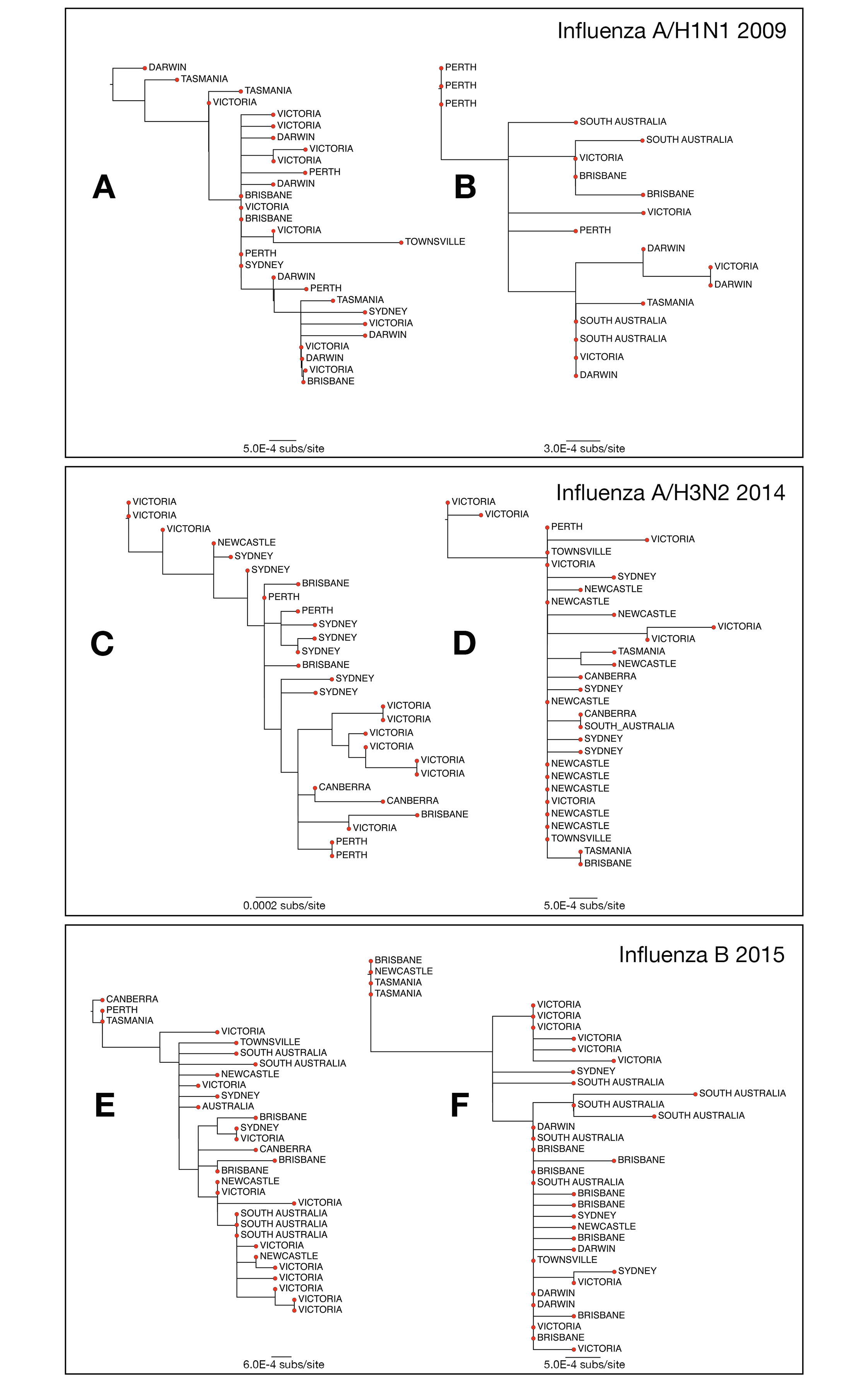

Supplement: S3 Fig — HA genetic sequences were downloaded from the GISAID EpiFlu database (platform.gisaid.org). Trees are rooted as in Fig 6. (TIF) [file ppat.1006780.s004.tif]
